# Imaging strategies of coarctation repairs

**DOI:** 10.1186/1532-429X-15-S1-T6

**Published:** 2013-01-30

**Authors:** Michelle F Walkden, Alison Fletcher, Charles Peebles, James Shambrook, Stephen Harden

**Affiliations:** 1 Cardiothoracic Radiology, University Hospital Trust Southampton, Southampton, UK

## Background

Coarctation is a common cardiovascular lesion accounting for 5 - 7% of all congenital heart disease. Without appropriate treatment complications are common and up to 90% of patients with uncorrected coarctation die by the age of 60.

After repair close follow-up of patients is recommended. Recognised complications may include residual or recurrent coarctation and aneurysm formation. Follow up of associated aortic valve disease is also required.

MRI is the preferred imaging modality for coarctation repairs as it provides anatomical and functional information without radiation exposure. It may also aid planning for further intervention or surgical treatment where necessary.

Available sequences;

Cine images- Steady State Free Precession (SSFP) or Gradient Recalled Echo (GRE) sequences, providing anatomical and functional information.

Black Blood Fast Spin Echo (FSE) provides good visualization of the vessel wall and is less susceptible to metal artefact and is therefore particularly useful following stenting.

Magnetic Resonance Angiography (MRA) either Contrast Enhanced or Free- breathing ECG triggered, navigator-gated 3D segmented SSFP. Both provide a 3D volume of data. This may be reformatted to any plane.

Phase Contrast imaging allows for assessment of blood flow and further physiological evaluation.

## Methods

In our dedicated Cardiac MR (CMR) unit, we have retrospectively evaluated studies from the past 6 years, where patients have had coarctation repair. With particular emphasis on the sequences used in relation to the type of repair. We have also looked at associated features of the aortic valve and left ventricle.

## Results

We will present a comprehensive imaging guide, highlighting the benefits and deficiencies of the MR sequences used in coarctation repairs, including common complications.

Key learning points;

Black Blood (FSE) and GRE are less susceptible to metal artefact therefore improve visualization around the stent.

CE-MRA or Navigator SSFP may be particularly useful in visualising aneurysm formation or extra cardiac conduits.

## Conclusions

Cardiac MR can provide excellent anatomical and functional information about coarctation repairs, providing imaging strategies are related to the type of repair done.

## Funding

No funding.

